# The Stigma of self-report in health research: Time to reconsider what counts as “Objective”

**DOI:** 10.1371/journal.pgph.0005521

**Published:** 2026-01-28

**Authors:** Nisreen A. Alwan

**Affiliations:** 1 School of Primary Care, Population Sciences and Medical Education, Faculty of Medicine, University of Southampton, Southampton, United Kingdom; 2 University Hospital Southampton NHS Foundation Trust, Southampton, United Kingdom; PLOS: Public Library of Science, UNITED STATES OF AMERICA

In quantitative health research, the term “self-report” often carries powerful stigma. It’s frequently treated as a second-class source of data—often automatically considered as more biased and less reliable than administrative health records. But such rigid hierarchy is not just flawed; it is harmful. It marginalizes the very people whose experiences we aim to understand and improve. If we truly want to advance health equity, we must destigmatise self-reporting, especially for health conditions that lack definitive diagnostic tools.

Self-reported data are routinely labelled as ‘subjective’. For years, I stood at the front of classrooms, extolling the virtues of the “objective” over the “subjective,” teaching students to prize “hard” data when examining causal relationships in epidemiology because self-reported data can be subject to bias. Such bias can manifest in different ways depending on whether the health outcome being studied is known at the time of exposure assessment. For instance, in a case-control study investigating the relationship of dietary patterns with cancer, individuals with cancer may recall their past dietary habits differently, introducing differential bias. In contrast, prospective cohort studies—such as one that tracks dietary supplement intake during pregnancy—may encounter non-differential bias due to general inaccuracies in reporting frequency, timing and type of supplements, irrespective of the future health outcome being studied.

However, the COVID-19 pandemic taught me to be more critical of blindly adopting such perspective when during its early stages I found myself in a paradoxical position. I was a public health academic whose own lived experience of Long Covid was invisible to our public health systems [1–3]. Like thousands of others who were suffering the long-term health effects of the virus and were not hospitalized, tested or diagnosed, we were not counted. Our experiences were only visible through self-report but that was commonly dismissed by the scientific establishment as anecdotal or less worthy of attention.

In researching health conditions like Long Covid with loose and variable case definitions and no specific diagnostics, self-reported answers are still frequently considered less valid than using healthcare records of clinical diagnostic codes. Symptoms -by definition- are only assessed by self-reporting. They cannot be ‘objectified’ by clinical examination or medical investigations. These so-called “objective” measures are supplementary at best. However, when someone says they have Long Covid, doubt creeps in—not because of evidence, but because of stigma [4].

Such stigma is not just a theoretical concern. It is a common barrier that may deter people from seeking recognition, help and care, or influence health professionals and employers in their judgement to provide them [5,6]. About one third of people with Long Covid regretted sharing their condition, and almost two thirds were “very careful” about whom they told [5]. Such self-censorship may be driven by fear of disbelief, dismissal, or discrimination. It can become a source of isolation and a strong driver of inequity.

Self-reported data are routinely undervalued for some other health conditions, and their dismissal is not unique to Long COVID. Menopausal symptoms, for example, are frequently not taken seriously when reported by women experiencing them. Menopausal symptoms can be ‘treated as a joke’ or women could be accused of ‘faking’ their symptoms or of ‘not being in their right mind’ [7,8]. But what other way can there be to ascertain if a woman is experiencing menopausal symptoms than self-report?

The assumption is that people misremember, exaggerate, or misinterpret their symptoms. But what if the problem lies not in the type of data, but in our unwillingness to trust this mode of reporting without adequate justification for such mistrust? This self-report prejudice can shape who gets counted, whose suffering is recognized, and whose needs are addressed. When we dismiss self-report, we risk silencing those who are already underserved.

This brings us to epistemic injustice—a concept coined by Miranda Fricker. It’s the idea that some people are wronged specifically in their capacity as knowers, and this can manifest in two forms: hermeneutical and testimonial [9]. In Long Covid, we see both “hermeneutical injustice”—where the lack of shared frameworks for a health condition makes peoples’ experiences hard to articulate to others or understand even by themselves—and “testimonial injustice,” where someone’s credibility is undermined by identity-based prejudice.

Epistemic injustice is evidenced in the qualitative data discussing the lived experience of people with Long Covid [6,10,11]. Quantitative analysis of the GP Patient Survey data (around 760,000 patients in England) shows that there were more people unsure about having Long Covid (hermeneutical injustice) than those who were (~9% v 5%) [12]. There were also variations in both the prevalence of Long Covid and in the status of being ‘not sure’ by identity characteristics such as gender, ethnicity, sexual orientation, being a carer, employment, and religion (testimonial injustice) [12].

**Fig 1:**
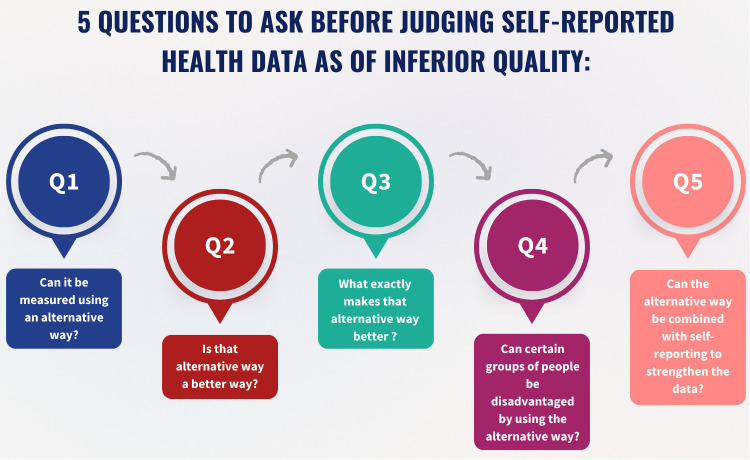
Five questions to ask before judging self-reported data as of inferior quality in quantitative health research.

To move forward, we must shift our misconception of self-report as a ‘last resort’ for data and reframe it as a cornerstone of inclusive research to understand the true burden of disease. This means challenging the epistemic hierarchy that automatically labels it as inherently flawed and unconditionally places biomedical data at the top and lived experience at the bottom. Critical assessment of bias needs to be contextual taking account of the nature of the health condition and the accuracy of the available methods to ascertain it (Fig 1).

Like any data source, self-report has limitations, but it also offers unique strengths. Health research is about centring people’s experiences. This is not just a matter of ethics; it’s a matter of validity. Importantly, as health professionals and researchers, we must acknowledge the courage it takes to self-report, especially in a system that judges it as inferior to other forms of ascertainment. We must de-stigmatise ‘self-reporting’ particularly for health conditions that are under-recognised, under-researched and disproportionately affected by epistemic injustice. We have a responsibility to truly listen, contextualise, and act.

It’s time to scrutinise the easy claims of “objectivity” in quantitative health research that too often sideline those most disadvantaged and widen health inequalities. Our pursuit of rigour should not perpetuate epistemic injustice, driving research further from the lived realities it seeks to understand.
